# Lipid Profile, PCSK9, ANGPTL3 and Lipoprotein (a) Levels in Men Diagnosed With Localized High‐Grade Prostate Cancer and Men At‐Risk of Prostate Cancer

**DOI:** 10.1002/cam4.70587

**Published:** 2025-01-31

**Authors:** Ann‐Charlotte Bergeron, Emilie Wong‐Chong, France‐Hélène Joncas, Chloé Castonguay, Frédéric Calon, Nabil G. Seidah, Jonatan Blais, Karine Robitaille, Alain Bergeron, Vincent Fradet, Anne Gangloff

**Affiliations:** ^1^ Oncology Research Axis, Centre de Recherche du CHU de Québec‐Université Laval Quebec City Quebec Canada; ^2^ Division of Molecular Medicine, Faculty of Medicine Université Laval Quebec City Quebec Canada; ^3^ Cancer Research Center (CRC) Université Laval Quebec City Quebec Canada; ^4^ Institute of Nutrition and Functional Foods (INAF) and NUTRISS Center ‐ Nutrition Health and Society of Université Laval Quebec City Quebec Canada; ^5^ Neuroscience Research Axis, Centre de Recherche du CHU de Québec‐Université Laval Quebec City Quebec Canada; ^6^ Laboratory of Biochemical Neuroendocrinology Institut de Recherches Cliniques de Montréal Montreal Quebec Canada; ^7^ Department of Surgery CHU de Québec‐Université Laval, Université Laval Quebec City Quebec Canada; ^8^ Department of Molecular Biology, Medical Biochemistry and Pathology CHU de Québec‐Université Laval, Université Laval Quebec City Quebec Canada

**Keywords:** ANGPTL3, lipid‐lowering drugs targets, lipoprotein (a), PCSK9, prostate cancer

## Abstract

**Background:**

Some cancers have been found to require abundant supplies of lipids for their development. One example is prostate cancer (PCa). To date, lipid‐modifying factors, such as proprotein convertase subtilisin/kexin type 9 (PCSK9), angiopoietin‐like 3 protein (ANGPTL3), and lipoprotein(a) or Lp(a), have not been reported in men with PCa. The present study aimed to verify whether plasma levels of these lipid‐related proteins vary in men with PCa compared to at‐risk but cancer‐free men.

**Methods:**

Plasma samples from 35 men with locally advanced PCa Gleason 8 and 9 versus 35 men at risk of PCa were selected as cases and controls. Blood samples were paired according to age and BMI. Apolipoprotein B100 (Apo B), Lp(a), and lipid profiles were measured on an analytical platform (Roche Cobas). PCSK9 and ANGPTL3 levels were determined by ELISA.

**Results:**

No significant change in lipids and related factors levels was observed between men with localized PCa Gleason 8 or 9 and matched controls. A correlation between ANGPTL3 and HDL levels was only confirmed in controls (ρ = 0.54, *p* = 0.0009). PCSK9 was inversely associated with PSA levels in the entire cohort (ρ = −0.31, *p* < 0.01), suggesting that factors influencing PCSK9 could also influence PSA levels. In controls only, PSA levels were correlated with LDL, Apo B, non‐HDL, total cholesterol, and triglycerides (all ρ coefficients ≥ 0.35, all *p*‐values < 0.05). PCSK9 was correlated to LDL in PCa men, but the relationship was unexpectedly found to be inverse.

**Conclusions:**

In this observational study, lipid profiles, PCSK9, ANGPTL3, and Lp(a) levels did not change in men diagnosed with locally advanced Gleason 8 or 9 PCa compared to at‐risk but cancer‐free men. The present data suggest a complex interplay between PCSK9, PSA, and the lipid profile in localized PCa.

## Graphical abstract

Measured prostate‐specific antigen (PSA) levels are correlated with the lipid profile and ANGPTL3 levels in men at risk for prostate cancer but no longer display this correlation in men diagnosed with prostate cancer. PCSK9 and PSA levels appear to be inversely correlated in the entire study cohort, gathering men at risk and men with prostate cancer.

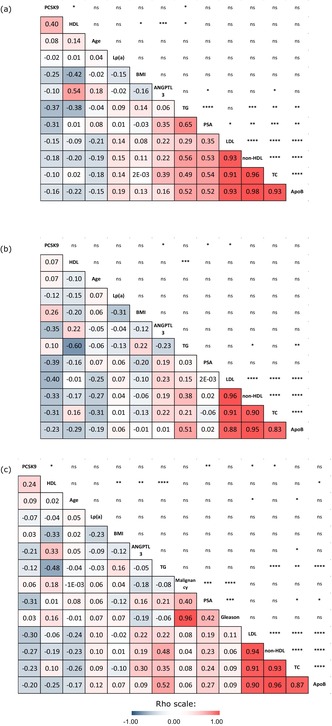

## 1 Introduction

Cancer is the leading cause of death in Canada [1] and a major cause of premature death worldwide [2]. Lipids, particularly cholesterol, are crucial in tumorigenesis by contributing to the formation of the cellular membranes in proliferating cells [3, 4, 5]. Lipids also contribute to carcinogenesis through steroid hormones synthesis [6, 7], inflammation [8], regulation of apoptosis [9, 10] and cell adhesion [11], highlighting their multifaceted role beyond serving as building blocks of cell membranes.

The relationship between cholesterol levels and prostate cancer (PCa) reported to date is conflicting, indicating the need to investigate further why positive [12, 13, 14], negative [15, 16], and absence of association [17, 18] have all been described. Many changes in lipid metabolism linked to PCa progression occur (e.g., lipoprotein receptor overexpression, changes in cholesterol efflux [19, 20, 21]), while cholesterol‐lowering drugs decrease PCa aggressiveness and mortality [22, 23].

New lipid‐lowering drugs targeting circulating PCSK9, ANGPTL3, and Lp(a) allow cholesterol levels to be lower than those attainable with statins or ezetimibe alone and are now available to treat cardiovascular diseases [24]. These factors could represent new targets in the treatment of PCa and need to be further explored. The present report centers on these three lipid‐related factors, their variations in men with high‐grade Gleason 8 or 9 localized PCa, and their effect on circulating lipids, as reviewed below.

Proprotein convertase subtilisin/kexin type 9 (PCSK9) regulates low‐density lipoprotein cholesterol (LDL‐cholesterol) levels by binding to LDL receptors and targeting them towards degradation, which leads to higher cholesterol levels. In turn, PCSK9 inhibition is used in cardiovascular protection to lower cholesterolemia [25, 26]. PCSK9 levels were already found to be elevated in patients with breast cancer [27] and non‐small cell lung cancer [28]. In mice with melanoma, PCSK9 deficiency has been shown to reduce hepatic metastasis [29]. PCSK9 could also decrease the expression of the major histocompatibility complex class I (MHC‐I) protein on the surface of tumors, thus preventing the infiltration of cytotoxic CD8^+^ T‐lymphocytes in the tumoral microenvironment [30].

Angiopoietin‐like protein 3 (ANGPTL3) contributes to lipid regulation by inhibiting lipoprotein lipase and endothelial lipase activities [31, 32, 33], increasing LDL, HDL and triglyceride levels. As such, inhibitors have been developed in the cardiovascular field. On the cancer side, in vitro and in vivo downregulations of *ANGPTL3* have been described in different models of oral squamous cancer [34], colorectal cancer [35], hepatocellular carcinoma [36] and epithelial high‐grade serous ovarian cancer [37], positioning ANGPTL3 and its inhibition as a promising target against cancer.

Lipoprotein (a), designated as Lp(a), is an LDL‐like particle with pro‐atherogenic and pro‐thrombotic effects conveyed by its pro‐inflammatory apolipoprotein (a) [38]. The relationship between cancer and Lp(a) is unclear, as studies come to inconsistent conclusions concerning Lp(a) pro‐ or anti‐neoplastic effects [39, 40, 41, 42, 43, 44, 45]. Genetically proxied Lp(a) levels were associated with total, advanced, as well as early‐age onset PCa (< 55 years old) by Mendelian randomization [46], making Lp(a) a circulating factor of interest in the present study.

Whether PCSK9, ANGPTL3 and/or Lp(a) play a role in PCa‐induced lipid metabolism reprogramming is an unresolved question. The present study aims to determine whether PCSK9, ANGPTL3 and Lp(a) levels are altered in patients with Gleason 8 and 9 locally advanced PCa.

## 2 Patients and Methods

### 2.1 Selection of Plasma Samples

The present protocol received approval from the ethics committee of the CHU de Québec‐Université Laval (Project# 2023–6537). We obtained 35 plasma samples from men with high‐grade localized PCa (Gleason score of 8 or 9 on biopsy) from the URO‐1 biobank. Thirty‐five plasma samples from cancer‐free men, investigated for PCa based on an elevated PSA level or a positive family history of PCa, were selected as controls from the BIOCaPPE‐GRePEC biobank. To assess elevated PSA levels, the following laboratory reference intervals for PSA (in ng/mL) were used: 20–29 years: < 1.10; 30–39 years: < 1.50; 40–49 years: < 2.00; 50–59 years: < 3.00; 60–69 years: < 4.00; > 70 years: < 6.00. These participants had a negative prostate biopsy and remained cancer‐free for at least 3.2 years after the biopsy and blood sampling. Controls were individually matched to cases based on age and body mass index (BMI). They were considered a match if they were 5 years or less apart and if their BMI belonged to the same category: normal, overweight, obesity class I, obesity class II, obesity class III.

Blood samples were drawn from participants between 2010 and 2019 in vacutainers containing EDTA. Plasma samples were obtained by centrifugation of whole blood from which plasma was separated and then kept at a temperature of −80°C, according to stringent institutional SOPs. They were thawed only once: on the day all assays were performed. The biobanks provided the following associated data: sex, age, BMI, PSA levels, tumor staging, TNM score, medication (alpha‐blockers and 5‐alpha‐reductase inhibitors), and occurrence of biochemical recurrence. The data were collected at the time of blood sampling, except for the biochemical recurrence, which was determined during follow‐up. Both biobanks (URO‐1 and BIOCaPPE‐GRePEC) were constituted with approval from the ethics committee of the CHU de Québec‐Université Laval (Projects #2012‐1002 and #2012‐1111, respectively). Consent was obtained from each participant prior to blood sampling and data collection.

### 2.2 Assessment of Apo B, Lp(a) and Lipid Profile

Apolipoprotein B100 levels (Apo B, g/L), Lipoprotein(a) levels (Lp(a), nmol/L), and lipid profile were measured on a Modular analytical platform (Roche Cobas) at the Core laboratory of the *Hôpital de l'Enfant‐Jésus* (Quebec City, Canada). Assessment of the lipid profile included measurements of total cholesterol (TC, mmol/L), HDL cholesterol (HDL, mmol/L), and triglycerides (TG, mmol/L). LDL‐cholesterol levels (LDL, mmol/L) were calculated with the Friedewald equation [47]: LDL = TC—(TG/2.2)—HDL. Non‐HDL cholesterol levels (non‐HDL, mmol/L) were calculated using the formula: non‐HDL = TC – HDL. Lp(a) measurements that were outside of the reportable range (< 10 nmol/L) were set to a default value of 3 nmol/L (½ of the lower limit of detection).

### 2.3 Assessment of PCSK9 and ANGPTL3 Levels by ELISA

PCSK9 and ANGPTL3 levels were quantified by enzyme‐linked immunosorbent assay (ELISA). The Legend Max ELISA kit (#443107, Biolegend, San Diego, CA, USA) and the SimpleStep ELISA kit (#ab254510, Abcam, Cambridge, MA, USA) were used for the measurement of PCSK9 and ANGPTL3, respectively, and according to manufacturers' instructions. Samples were diluted 1:30 for PCSK9 and 1:50 for ANGPTL3. All samples were measured in duplicates on the same day. Variability between samples assayed in duplicates (intra‐assay coefficient of variability and standard deviation or CV ± SD) averaged 3.00% ± 2.60% for PCSK9 and 3.77% ± 3.27% for ANGPTL3. Assay‐to‐assay variability between ELISA plates (inter‐assay CV ± SD) averaged 11.04% ± 11.79% for PCSK9 and 3.14% ± 0.45% for ANGPTL3.

### 2.4 Statistical Analyses

A minimal sample size of 35 individuals per group for a statistical power of 80% and an alpha (α) error of 0.05 was estimated a priori with G*Power software version 3.1. All other statistical analyses were conducted using R software version 4.2.1, admitting a type I error α = 0.05. Significance thresholds were as follows: *p < 0.05, **p < 0.01, ***p < 0.001, ****p < 0.0001. Shapiro–Wilk Normality tests were performed on numerical variables to assess the normality (Gaussian character) of their distribution. Two‐sided Mann–Whitney–Wilcoxon tests (for non‐normally distributed datasets) and unpaired Student's *t*‐tests (for normally distributed datasets) were used to assess differences between controls and cases in age, BMI, PSA, triglycerides, total cholesterol, HDL, non‐HDL, LDL, Apo B, Lp(a), ANGPTL3, and PCSK9. After matching cases and controls by age and BMI, two‐sided Wilcoxon Signed Rank tests and paired Student's *t*‐tests were performed for each variable, and respectively for non‐normally and normally distributed pair differences. Pairwise correlation analyses were performed on the control, PCa, and in the whole cohort to assess the association between variables based on Spearman's rank order tests, admitting a two‐tailed hypothesis. The Corx package (courtesy of Dr. James Conigrave, https://github.com/conig/corx) was used to compute matrices of rho coefficients as estimates of the correlation strength. The pcor.test() function from the ppcor R package was used to compute standard and partial correlation coefficients and their precise p‐values.

## 3 Results

### 3.1 Study Population Characteristics

Characteristics of the study population, including the results of two‐group comparisons, are presented in Table 1. All blood samples were drawn from fasting men, except for one participant in each group who was non‐fasting. Those two samples were from non‐fasting participants not part of the same matching pair. Both remained within a range defined by the mean ± 1 SD for the entire cohort and their respective group, except for the following variables: a non‐HDL level of 2 mmol/L and an Lp(a) level of 164 nmol/L in the non‐fasting control; a PCSK9 level of 145 ng/mL in the non‐fasting case. Nearly all cases were matched to a control of the same age, except for two pairs with a difference of 4 years (73 vs. 69 years old) and 5 years (74 vs. 79 years old). The maximal difference in BMI between participants of the same match was 3.25 kg/m^2^.

**TABLE 1:** Characteristics of the study population.

	Men at risk (*n* = 35)	Men with prostate cancer, (*n* = 35)	Total (*n* = 70)	*p* (unpaired test)^a^	*p* (paired test)^a^
Gleason score					
No cancer	35 (100%)	0 (0%)	35 (50.0%)		
Gleason 8	0 (0%)	26 (74.3%)	26 (37.1%)		
Gleason 9	0 (0%)	9 (25.7%)	9 (12.9%)		
Primary tumor staging					
T2	0 (0%)	12 (34.3%)	12 (17.1%)		
T3	0 (0%)	20 (57.1%)	20 (28.6%)		
None	35 (100%)	3 (8.6%)	38 (54.3%)		
Lymph node involvement					
N0	0 (0%)	25 (71.4%)	25 (35.7%)		
N1	0 (0%)	7 (20.0%)	7 (10.0%)		
None	35 (100%)	3 (8.6%)	38 (54.3%)		
Biochemical recurrence					
No	0 (0%)	12 (34.3%)	12 (17.1%)		
Yes	0 (0%)	15 (42.9%)	15 (21.4%)		
None	35 (100%)	8 (22.9%)	43 (61.4%)		
Age (years)					
Mean (SD)	66.7 (7.36)	66.7 (7.49)	66.7 (7.37)	1.0	1.0
Median [min–max]	67.0 [50.0–77.0]	67.0 [50.0–79.0]	67.0 [50.0–79.0]		
BMI (kg/m^2^)					
Mean (SD)	28.5 (3.78)	28.7 (4.08)	28.6 (3.90)	0.77	0.39
Median [min–max]	28.3 [21.4–40.6]	28.5 [20.7–41.0]	28.5 [20.7–41.0]		
Normal (18.5–24.9)	4 (11%)	4 (11%)	8 (11%)		
Overweight (25–29.9)	20 (57%)	20 (57%)	40 (57%)		
Obesity class I (30–34.9)	9 (26%)	9 (26%)	18 (26%)		
Obesity class II (35–39.9)	1 (3%)	1 (3%)	2 (3%)		
Obesity class III (≥ 40)	1 (3%)	1 (3%)	2 (3%)		
PSA (ng/mL)					
Mean (SD)	5.77 (4.75)	13.0 (12.4)	9.38 (10.0)	0.001***	0.002**
Median [min–max]	5.38 [0.190–28.0]	8.40 [0.320–56.0]	6.35 [0.190–56.0]		
Total cholesterol (mmol/L)					
Mean (SD)	4.51 (1.17)	4.64 (0.939)	4.57 (1.06)	0.52	0.62
Median [min–max]	4.32 [2.49–7.16]	4.58 [3.31–6.48]	4.43 [2.49–7.16]		
HDL (mmol/L)					
Mean (SD)	1.19 (0.288)	1.31 (0.369)	1.25 (0.335)	0.14	0.12
Median [min–max]	1.13 [0.750–1.90]	1.22 [0.830–2.89]	1.19 [0.750–2.89]		
Triglycerides (mmol/L)					
Mean (SD)	1.70 (1.19)	1.47 (0.660)	1.59 (0.960)	0.52	0.37
Median [min–max]	1.36 [0.700–7.69]	1.28 [0.510–3.36]	1.36 [0.510–7.69]		
LDL (mmol/L)					
Mean (SD)	2.55 (1.00)	2.67 (0.867)	2.61 (0.933)	0.51	0.61
Median [min–max]	2.25 [0.900–4.92]	2.50 [1.48–4.56]	2.43 [0.900–4.92]		
Apolipoprotein B (g/L)					
Mean (SD)	0.859 (0.284)	0.868 (0.220)	0.863 (0.253)	0.60	0.89
Median [min–max]	0.790 [0.370–1.53]	0.810 [0.500–1.40]	0.800 [0.370–1.53]		
Non‐HDL (mmol/L)					
Mean (SD)	3.32 (1.23)	3.33 (0.988)	3.33 (1.10)	0.78	0.96
Median [min–max]	3.08 [1.25–6.29]	3.14 [1.82–5.46]	3.10 [1.25–6.29]		
Lipoprotein(a) (nmol/L)					
Mean (SD)	57.1 (70.4)	84.5 (108)	70.8 (91.8)	0.64	0.68
Median [min–max]	25.0 [3.00–237]	23.0 [3.00–346]	24.0 [3.00–346]		
ANGPTL3 (ng/mL)					
Mean (SD)	36.3 (17.0)	32.2 (18.9)	34.3 (18.0)	0.14	0.051
Median [min–max]	36.8 [11.6–86.6]	27.3 [13.3–115]	29.1 [11.6–115]		
PCSK9 (ng/mL)					
Mean (SD)	84.8 (23.3)	88.8 (27.8)	86.8 (25.5)	0.52	0.58
Median [min–max]	84.5 [28.8–152]	83.4 [39.9–145]	84.0 [28.8–152]		

*Note:* a The Mann–Whitney‐Wilcoxon test was used for unpaired group comparison. For PCSK9, data were normally distributed and a Student's *t*‐test with Welch correction for unequal variances was performed instead. The following variables, for which pair differences were non‐normally distributed, required a Wilcoxon Signed Rank test for paired group comparison: age, BMI, PSA, triglycerides, Lp(a) and ANGPTL3. Otherwise, paired Student's *t*‐test were used *p < 0.05 **p < 0.01 ***p < 0.001.

Six participants from the PCa group were taking 5‐alpha‐reductase inhibitors. Two participants from the PCa group and one participant from the control group were taking alpha‐blockers. As expected, PSA levels were lower in the control group (mean ± SD = 5.77 ± 4.75 ng/mL, median = 5.38 ng/mL) than in the PCa group.

### 3.2 Lipid Profile and Lipid‐Related Factors Between Groups

**FIGURE 1:**
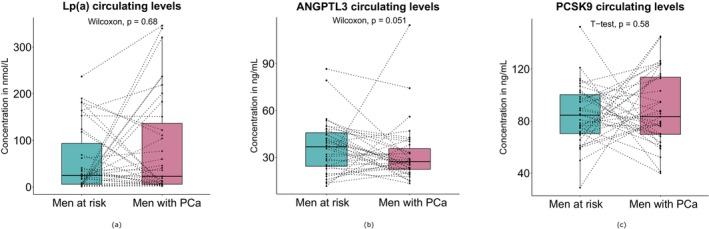
Circulating levels of (a) Lp(a), (b) ANGPTL3, and (c) PCSK9 between men at risk (without PCa) and men with prostate cancer (PCa), using boxplots. Dotted lines connect individuals who have been age‐matched and BMI‐matched. A Wilcoxon Signed Rank test was performed to compare variables with non‐Gaussian distribution. A paired Student's *t*‐test was performed on variables showing normal distribution.

Triglycerides, total cholesterol, HDL, non‐HDL, LDL, and Apo B levels were similar in the two groups. Lp(a), ANGPTL3, and PCSK9 levels did not differ between groups (Table 1; Figure 1a–c). Lp(a) levels showed a tendency to increase in the PCa group albeit non‐significantly (Table 1, means ± SD of 84.5 ± 108 in cases vs. 57.1 ± 70.4 nmol/L in men at risk, medians of 23.0 in cases vs. 25.0 nmol/L in men at risk). ANGPTL3 levels (Figure 1b) were slightly lower in the PCa group, particularly in the case of paired comparisons. Still, this difference did not reach statistical significance (Table 1, means ± SD: 32.2 ± 18.9 vs. 36.3 ± 17.0 ng/mL, medians: 27.3 vs. 36.8 ng/mL, Mann–Whitney–Wilcoxon test *p*‐value of 0.14, Wilcoxon signed rank test *p*‐value of 0.051).

### 3.3 Correlation Analyses

**FIGURE 2:**
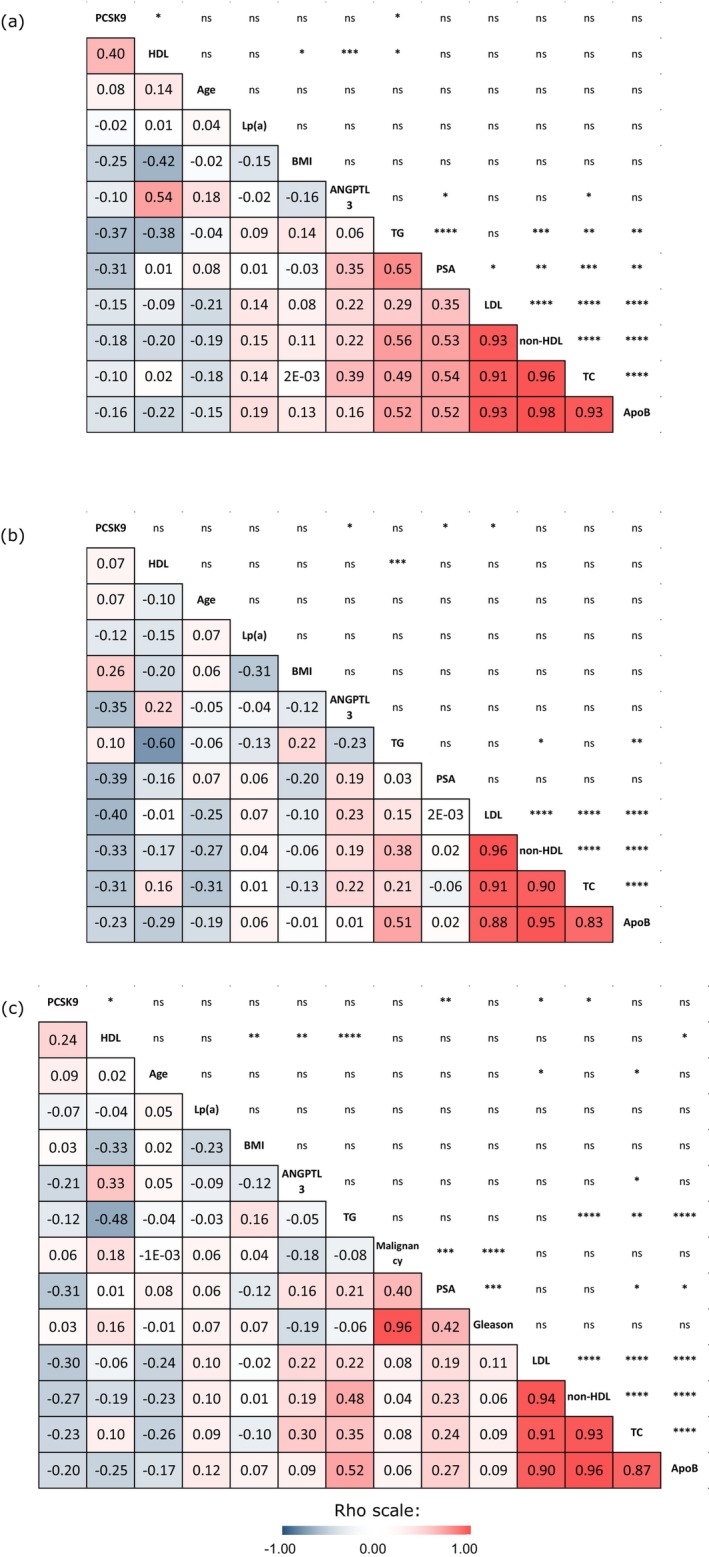
Matrices of pairwise correlation for (a) men at risk, (b) men with prostate cancer and (c) the entire cohort. Matrices were computed based on Spearman's rank order. Lower half‐panel: Rho coefficients. Upper half‐panel: Significance levels: *p < 0.05, **p < 0.01, ***p < 0.001, ****p < 0.0001. Color shades indicate correlation strength estimated with rho coefficients; the darker the color, the stronger the association. Blue‐shaded values are representative of negative associations, while red‐shaded values depict positive associations (see rho coefficient scale). Rho coefficients of −1 or 1 indicate perfect negative or perfect positive correlation. A rho coefficient of 0 defines a total absence of correlation between the two variables considered. TC, total cholesterol; TG, triglycerides.

To investigate whether localized high‐grade prostate cancer (PCa) modifies existing links between lipids and their related factors under study, Spearman's rank‐order tests were performed. Estimates of correlation strength between all variables are displayed as rho correlation coefficients in Figure 2.

#### 3.3.1 Association Between Circulating Lipids

Atherogenic particles (total cholesterol, Apo B, non‐HDL, and LDL) displayed strong pairwise correlations with one another, as illustrated by Spearman's ρ coefficients ≥ 0.83 with *p*‐values *p* < 0.0001 across all groups (Figure 2a–c). HDL levels were inversely correlated with triglycerides in the control group (Figure 2a, ρ = −0.38, *p* = 0.024), the PCa group (Figure 2b, ρ = −0.60, *p* = 0.00014), and the whole cohort (Figure 2c, ρ = −0.48, *p* = 2.7 × 10^−5^), which are well‐documented and expected associations.

#### 3.3.2 PSA Association With ANGPTL3, Lipids and PCSK9

In the control group (Figure 2a), strong positive correlations were observed between each type of atherogenic particle and PSA. Spearman's rank order tests provided the following correlations with PSA: ANGPTL3 (ρ = 0.35, *p* = 0.042), triglycerides (ρ = 0.65, *p* = 2.65 × 10^−5^), total cholesterol (ρ = 0.54, *p* = 0.00076), LDL (ρ = 0.35, *p* = 0.038), Apo B (ρ = 0.52, *p* = 0.0015) and non‐HDL (ρ = 0.53, *p* = 0.0010). These correlations weakened markedly, becoming non‐significant in the PCa group (Figure 2b). PCSK9 levels displayed a modest but consistent negative correlation with PSA in the whole cohort (Figure 2c, ρ = −0.31, *p* = 0.0084) and the PCa group (Figure 2b, ρ = −0.39, *p* = 0.022). The negative correlation between PSA and PCSK9 did not reach statistical significance in the control group (Figure 2a, ρ = −0.31, *p* = 0.07).

#### 3.3.3 Association Between PCSK9, ANGPTL3 and the Lipid Panel

The well‐documented negative correlation linked PCSK9 and LDL in the PCa group (Figure 2b, ρ = −0.40, *p* = 0.016) and the whole cohort (Figure 2c, ρ = −0.30, *p* = 0.011) but not in the control group (Figure 2a, ρ = −0.15, *p* = 0.57). PCSK9 levels in the control group (Figure 2a) were positively correlated with HDL levels (ρ = 0.40, p = 0.016) and negatively correlated with triglyceride levels (ρ = −0.37, *p* = 0.027). These correlations disappeared in the PCa group (Figure 2b, ρ = 0.07 and *p* = 0.71 for PCSK9 & HDL; ρ = 0.10 and *p* = 0.57 for PCSK9 and triglycerides). Increased PCSK9 levels were significantly associated with decreased ANGPTL3 levels in the PCa group only (Figure 2b, ρ = −0.35, *p* = 0.04). Increased ANGPTL3 was associated with increased levels of total cholesterol (ρ = 0.39, *p* = 0.021) and HDL (ρ = 0.54, *p* = 0.00091) in the control group (Figure 2a), but not in the PCa group (Figure 2b).

## 4 Discussion

The objective of the present study was to measure changes in lipid‐related factors that may be induced by the presence of prostate cancer Gleason 8 or 9. To that end, levels of PCSK9, ANGPTL3, Lp(a), and lipids were measured in two groups of men. Men in the first group had a confirmed diagnosis of localized high‐grade PCa. Men in the second group were at risk for PCa (elevated PSA levels or familial history of PCa) but had not developed the disease during a minimum follow‐up period of 3.2 years following the negative biopsy.

### 4.1 Main Findings

Three main findings arise from the present study. The first relates to the study's objectives. It indicates that lipid profiles, PCSK9, ANGPTL3, and Lp(a) levels were not significantly altered in localized high‐grade (Gleason 8 or 9) PCa patients compared to cancer‐free men. The second finding confirmed an association between the lipid profile and PSA levels. This association was no longer observed in PCa patients. Lastly, and for the first time, the present data report an association between PCSK9 and PSA levels. PCSK9 is a known hyperlipidemic factor, yet we report an unexpected inverse association with LDL in men with Gleason 8 or 9 localized PCa in the present study.

The current data suggest that a complex interplay between PCSK9, PSA, and the lipid profile may occur in localized high‐grade PCa, reflecting a compensating mechanism related to dynamic changes in cholesterol uptake by PCa.

### 4.2 Lipid Profile in Localized, Gleason 8 or 9, High‐Grade PCa

In the present study, lipid profiles were similar between men with and without PCa. Cholesterol metabolism has been reported to be dysregulated in prostate cancer. High circulating cholesterol has been found to increase the risk of aggressive prostate cancer [8]. Solomon and Freeman have suggested that high cholesterol could be a risk factor for PCa, while PCa itself could deplete cholesterol [48]. Similarly, low serum cholesterol has been reported to lower the risk of high‐grade prostate cancer in patients [12]. Cholesterol levels result from a balance between the exogenous and endogenous pathways, physiological and pathological processes, and medications. It is therefore not unexpected that the investigations into the relationship between cholesterol levels and prostate cancer (PCa) have reported conflicting findings. While past studies have evidenced positive [12, 13, 14], negative [15, 16], and absence of association [17, 18], the current study did not find a significant association between lipid profile and the pre‐metastatic disease stage.

Factors that could explain the discrepancies found in the literature include the precise stage of PCa at the time of the venipuncture and the use of lipid‐lowering drugs. In our study, comparisons between PCa and control groups did not indicate changes in triglycerides, total cholesterol, HDL, LDL, non‐HDL, and Apo B in men with localized high‐grade PCa compared to at‐risk men. The absence of observable changes between at‐risk men and those with Gleason 8 or 9 PCa does not preclude the possibility that changes in lipid profiles or lipid‐regulating factors may occur later, once the metastatic process is underway. Other variables to consider are changes in cancer cell lipid metabolic pathways demonstrated in vitro, such as lipoprotein receptor overexpression [19, 20] and enhanced cholesterol influx in PCa cell lines [21]; both could affect cholesterol levels and be implicated in the progression of PCa.

### 4.3 Lipoprotein (a) in Localized High‐Grade PCa

No Lp(a) level changes were observed between men with Gleason 8 or 9 PCa and the control group. Lp(a) seemed higher on average in the PCa group (Table 1; Figure 1a), but this trend was non‐significant. It was neither reflected by median Lp(a) values nor by two‐group comparisons (Table 1). The seemingly higher Lp(a) levels in the PCa group are likely driven by significant variability among these subjects. It is also possible that the small group size in the present study precludes the identification of a significant difference in Lp(a) levels between groups. The present results cannot confirm higher Lp(a) levels in PCa, as predicted by a recent Mendelian randomization study performed on a population notably different from the present study. The present study isolated locally advanced Gleason 8 and 9, non‐metastatic subjects, as opposed to men from the Mendelian randomization study who had advanced PCa defined as metastatic or Gleason score (GS) ≥ 8 or PSA > 100 ng/mL, or PCa death [46, 49]. Therefore, Lp (a) could still be associated with PCa in men at the metastatic stage.

### 4.4 ANGPTL3 In Localized PCa

Elevations of ANGPTL3 levels were reported in hepatocellular carcinoma [36] and high‐grade serous ovarian cancer [37]. ANGPTL3 tumoral expression was also upregulated in human high‐grade serous ovarian carcinoma [50] and colorectal cancer tissues [35], while they showed downregulation in renal cell carcinoma [51].

For the first time, we report circulating levels of ANGPTL3 in localized Gleason 8 or 9 high‐grade PCa. A tendency towards a decrease in ANGPTL3 was observed among men with localized PCa. This decrease was non‐significant, but close to being so in paired comparisons. Given the small size of the cohort, this trend should be explored in a larger cohort and in a sub‐group formed of only metastatic PCa men, providing adequate statistical power to detect small but significant variations and allowing the distinction between localized and metastatic high‐grade PCa.

A lack of change in ANGPTL3 levels suggests that these are not involved at the localized stage of PCa. ANGPTL3 is both a hyperlipidemic and an angiogenic factor, so it will be interesting to study its trends in a metastatic population, in which angiogenesis might play a more preeminent role.

ANGPTL3 levels were positively correlated with HDL levels as expected and described before [33, 52]. Interestingly, this association weakened considerably in men with localized PCa (Figure 2b). The same phenomenon was previously observed between cancer‐free women and women with ovarian cancer [37]. The presence of PCa in men, similarly to the presence of ovarian cancer in that prior study, seems to disrupt the association between ANGPTL3 and HDL levels.

ANGPTL3 is known for inhibiting lipoprotein and endothelial lipase, responsible for VLDL‐triglycerides hydrolysis in blood capillaries. Hence, ANGPTL3 contributes to higher circulating beta‐lipoproteins levels [32], which are available for tumor uptake. ANGPTL3 is also an angiogenic factor which can help tumors develop neovascularization [53, 54, 55]. Tumor‐induced variations of ANGPTL3 [56] and uptake of HDL‐cholesterol by cancer cells [57] have been reported, perhaps explaining the disruption between ANGPTL3 levels and HDL in ovarian and prostate cancers.

### 4.5 Correlation Between PSA, ANGPTL3 and Lipid Profile in Men at Risk

Previous studies have reported associations between PSA and lipid levels in men without PCa [58]. PSA is a protein all prostate cells produce and is commonly used to screen for PCa [59]. In the control group of the present study, PSA levels were correlated with ANGPTL3, triglycerides, total cholesterol, LDL, non‐HDL and Apo B levels (Figure 2a). Astonishingly, these associations were completely lost in the PCa group. We hypothesize that the loss of association between PSA and all the above‐mentioned variables is due to the changes in lipid metabolism induced by the presence of the localized Gleason 8 or 9 PCa tumor.

An association between PSA and cholesterol levels among cancer‐free men similarly to the one revealed herein was suggested in a study published in late 2008 [60]. In that longitudinal study of 1214 cancer‐free men, the use of statins and consequent lowering of LDL‐cholesterol levels were linked to a decrease in PSA levels by a median of 4.1%. In the same study, men with PSA values ≥ 2.5 ng/mL and experiencing > 41% decline in their LDL levels after starting statin showed a PSA decline of > 17%. Another study of 962 cancer‐free men in 2009 also supported this PSA‐cholesterol interaction [61].

Concerning PSA and triglyceride levels, we found two studies investigating the relationship between these two variables. Through multiple linear regression analysis, both studies demonstrated a negative association between PSA levels and triglycerides [62, 63]. Moreover, the former study [62], which focused on a male cohort of 6774 participants aged 20–49 with PSA levels < 4 ng/mL and unknown cancer status, showed no correlation between PSA and HDL or total cholesterol. The second study, pointing towards an inverse correlation between triglycerides and PSA [63], examined 2919 male participants aged > 40, excluding PCa patients. In contrast, our study revealed a strong positive correlation between PSA and triglycerides in cancer‐free men aged 50 to 77 (Figure 2a, ρ: 0.65; *p* < 0.0001).

Biopsies are largely prescribed based on PSA blood levels, which were previously shown to be influenced by lipid‐lowering medications in PCa‐free men [64]. Given the association between the lipid profile and PSA levels in the control group, a question having clinical implications must be raised: should lipid‐lowering drugs be transiently stopped before PSA testing in men being screened for PCa? Since blood lipids are associated with PSA levels, lipid‐lowering therapy could lead to bias in PCa risk‐stratification of these men. Clarifying the directionality of this interaction between lipids and PSA may prevent over‐ or under‐prescription of prostate biopsies.

### 4.6 Correlation Between PSA, PCSK9 and Other Lipid‐Related Factors

While no correlation between PSA and the lipid profile was observed in the PCa group (Figure 2b), an inverse association between PSA and PCSK9 remained consistent across groups (Figure 2a–c), the threshold for statistical significance, however, was not reached in the control group of men at risk (Figure 2a).

This inverse correlation between PSA and PCSK9 is worth exploring since both PSA and PCSK9 levels are linked to lipids. Adding participants with lower PSA levels (< 3 ng/mL) to the cohort would help clarify this association. Obtaining prostate biopsies to confirm the lack of PCa in individuals with low PSA is challenging since there is little indication for biopsies in this subgroup of men [65].

Contrary to the expected positive association, a significant inverse association between PCSK9 and LDL levels appeared in the PCa group (ρ = −0.40, *p* < 0.05; Figure 2b). The association between PCSK9 and LDL is known to be direct and proportional; not inversely proportional [66, 67]. PCSK9 is secreted by hepatocytes to act as a regulator of LDL‐receptors (LDLR) expression [26]. LDLR decreases LDL‐cholesterol by binding LDL particles and initiating the endocytosis of LDL‐cholesterol from the bloodstream. Therefore, higher PCSK9 leads to lower LDLRs and higher circulating LDL‐cholesterol levels [68]. Lower levels of PCSK9 are thus expected to lead to more LDLR expression at the hepatocyte surface, lowering LDL‐cholesterol levels [69].

A hypothesis we propose for explaining the unexpected inverse association observed between PCSK9 and LDL levels in PCa is an increase in production and release of PCSK9 by hepatocytes. The presumed purpose behind this PCSK9 response would be to re‐establish circulating levels of LDL consumed by tumor cells. Given that the current investigation has not revealed changes in LDL cholesterol in high‐grade localized PCa, dynamic changes in circulating LDL may be actively compensated by PCSK9. This assumption can be proposed but not verified solely based on the present correlation analyses. Therapies aimed at downregulating circulating LDL‐cholesterol levels (e.g., statins) increase PCSK9 levels, resulting in enhanced degradation of LDLR as reported in diseases other than cancer [68]. Consequently, by protecting hepatic LDLR, PCSK9 inhibitors may be particularly beneficial as an add‐on therapy for patients receiving standard LDL cholesterol‐lowering medications.

### 4.7 Alpha‐Blockers and 5‐Alpha‐Reductase Inhibitors

About 20% of patients in the prostate cancer group were using medications for their prostate, namely alpha‐blockers to ease symptoms of benign prostate hyperplasia (BPH) and 5‐alpha‐reductase inhibitors used to treat BPH and androgenic alopecia. Participants were on either or both medications, each with distinct effects on circulating lipids. Studies with healthy volunteers taking 5‐alpha‐reductase inhibitors vs. placebo showed no difference in circulating total cholesterol, LDL, HDL, and triglycerides [64, 70]. Alpha‐blockers could decrease blood levels of cholesterol and LDL, whereas HDL could increase [71, 72]. Given that medications affecting the lipid profile and used by the men in the present cohort belong mainly to the alpha‐blocker class, the impact on our results is limited, with only two participants in the cancer group and one in the control group taking alpha‐blockers. A reassuring observation is that most of their measurements were not significantly different from the group's mean (± 1 SD).

### 4.8 Study Limitations and Strengths

In the present study, we had no information on participants' ethnicity [58] or prior use of lipid‐lowering drugs [68], which could have impacted lipid profiles and PCSK9, ANGPTL3, Lp(a) levels, or PSA levels [58, 68]. A group of age‐matched, BMI‐matched men with no risk factors wasn't available. Still, it would arguably have made a better control group for assessing these circulating factors in PCa. The current control group consisted of men at risk for PCa (family history of PCa and/or elevated PSA) but who remained without cancer for a minimum follow‐up period of 3.2 years following a negative prostate biopsy. We cannot ensure that men in the control group did not develop cancer beyond this period. An underlying pre‐cancerous condition that could affect blood measurements in the control group is unlikely but cannot be ruled out. Yet, interesting distinctions have been found between cases and controls at risk, the most striking one being a strong relationship between PSA levels and the lipid profile in controls, absent from cases.

Strong correlations measured between beta‐lipoprotein markers (LDL, Apo B, and non‐HDL) and between triglycerides and HDL [73, 74] were both expected and observed, providing internal quality control and validating the methodology used to measure lipid panel and related factors. Measurement accuracy and quality of the analyses were also enabled by the reduction of confounding effects of age and BMI, which can both impact PCa occurrence and lipid metabolism.

The lack of association between some variables can be due to the small size of our cohort, which indicates the need to corroborate results in a larger cohort. On the other hand, small cohorts enable the detection of strong associations, such as the relationship between PSA and the lipid profile, which was disrupted in men with localized advanced PCa. The variables that did not display any changes in the cases‐controls group comparisons may still hold interest for further investigation in larger cohort of PCa men and in men with metastatic PCa. As such, the absence of changes in Lp(a), PCSK9 and ANGPTL3 levels between men with localized PCa and men at risk could depend on the investigated cancer stage. Metastatic PCa, which requires more lipids and neovascularization, might show differences in the lipid‐related factors under study and should be investigated.

## 5 Conclusions

No significant differences in lipid profiles, Lp(a), ANGPTL3, and PCSK9 levels were evidenced between men diagnosed with localized high‐grade Gleason 8 or 9 prostate cancer (PCa) compared to men at risk for PCa who remained cancer‐free for at least 3.2 years following a negative biopsy. This study confirms a relationship between PSA levels and the lipid profile in cancer‐free men; a relationship lost in men with localized advanced PCa. Moreover, this study raises the question of lipid‐lowering drugs effect on PSA measurement and its impact on PCa screening and risk‐stratification. Finally, this study reports for the first time a complex interplay between PSA, PCSK9 and the lipid profile in localized but advanced PCa, which should be confirmed in larger size studies.

## Author Contributions

**Ann‐Charlotte Bergeron:** data curation (lead), formal analysis (lead), software (equal), validation (equal), visualization (supporting), writing – original draft (lead). **Emilie Wong‐Chong:** data curation (supporting), formal analysis (supporting), methodology (supporting), software (supporting), validation (supporting), visualization (lead), writing – original draft (lead), writing – review and editing (lead). **France‐Hélène Joncas:** data curation (supporting), investigation (equal), methodology (lead), project administration (equal), supervision (equal), validation (equal). **Chloé Castonguay:** validation (equal), writing – review and editing (equal). **Frédéric Calon:** resources (supporting), validation (equal), writing – review and editing (equal). **Nabil G. Seidah:** validation (equal), writing – review and editing (equal). **Jonatan Blais:** validation (equal), writing – review and editing (equal). **Karine Robitaille:** data curation (equal), resources (supporting), validation (equal), writing – review and editing (equal). **Alain Bergeron:** resources (equal), validation (equal), writing – review and editing (equal). **Vincent Fradet:** resources (lead), validation (equal), writing – review and editing (equal). **Anne Gangloff:** conceptualization (lead), funding acquisition (lead), investigation (lead), methodology (lead), project administration (equal), resources (lead), supervision (equal), validation (equal), writing – original draft (lead).

## Ethics Statement

The study was conducted in accordance with the Declaration of Helsinki, and approved by the *Comité d'éthique de la recherche du CHU de Québec‐Université Laval* (protocol code 2023‐6537 on October 20, 2022).

## Conflicts of Interest

The authors declare no conflicts of interest.

## Data Availability

The data supporting this study's findings are available on request from the corresponding author. The data are not publicly available due to privacy or ethical restrictions.
